# Skin examination in extreme conditions

**DOI:** 10.3389/abp.2026.15045

**Published:** 2026-05-01

**Authors:** Joanna Igielska-Kalwat

**Affiliations:** Faculty of Food Science and Nutrition, Poznan University of Life Sciences, Poznan, Poland

**Keywords:** extreme condition, sebum level, skin hydration level, skin pH, skin reaction

## Abstract

Extreme indoor and outdoor conditions in patients’ places of stay (hot or cold air and changes in humidity) lead to weakening of the lipid barrier function and disproportionate epidermal microbiota which results in a lower threshold of physical and chemical tolerance of the skin manifesting as erythema, burning, dryness and deterioration of skin condition. Chronic epidermal irritation caused by the above factors leads to discomfort and prevents the proper functioning of the skin, as it results in thickening of the epidermis, widening of the sebaceous glands and pigmentation disorders. Regenerative mechanisms preventing lipid peroxidation or carbonylation of skin cell proteins, including epidermis, are focused on restoring physiology and not on “fibroblast protection”, which can accelerate the loss of firmness. Taking into account the theory of free radical or telomeric aging, it may be stated that the skin exposed to extreme conditions ages faster. When faced with such a problem, it seems pointless to apply invasive rejuvenating treatments without prior diagnosis or acquiring basic knowledge of care and hygiene of the epidermis. Daily skin care and cosmetics play a key role in the anti-aging process. Hygiene and proper cosmetic habits are essential for health and youthful appearance of the skin. Appropriate regulatory exfoliation, or the so-called turnover of the epidermis, should be used so as not to generate excessive free radical stress. Antioxidants are necessary for this. Modern products based on a liquid crystal base compatible with the liquid crystal structure of epidermal cement together with active emollients allow for restoration of appropriate epidermis parameters. The method of exfoliation with selected ingredients enables long-term skin cleansing by accelerating the turnover of the epidermis without excessive free radical stress, drying and irritation.

## Introduction

The stratum corneum is the main barrier that protects the body against the penetration of various types of chemical compounds, insects, radiation, bacteria and gases. It also protects the body against too high or low temperature, and retains liquids and mineral salts inside it. This organ is a self-renewable system of alternating cells of the epidermis. Skin performs also a very important excretory role for unnecessary waste products. Dermis is the internal structure of the skin made up of a reticulate and papillary layer connected directly to the epidermis. This layer, in addition to the functions responsible for physical resistance and flexibility, conditions the nutrition of the epidermis. The formation of the epidermal barrier is a mechanism of proliferation of individual layers of the epidermis which later undergo keratosis and together with lipids form a protective corneous structure. This process can be divided into several stages: Keratin and lipid formation, granular layer and keratosis (Hui-Beckman et al., 2023). During the process of loss of keratinocyte enzymatic function, flat and thin structures of dead cells are formed in layers. Numerous lamellar bodies appear between the corneocytes and the hydrophilic and hydrophobic components are able to penetrate the epidermis. It is a process conditioning the reconstruction of the lipid coat. Particularly important for the protective function of the epidermis is the proper production of so-called cornified envelope resulting from terminal corneocyte apoptosis and cross-linking by transglutaminases. The main role in this process is played by filaggrin - FLG (filament aggregation protein), an alkaline stratum corneum protein binding keratin fibers. Filaggrin is synthesized from the so-called profilaggrin within the granular layer of the epidermis, in lamellar bodies. The ultimate corneocyte differentiation is also attributed to the activity of proteins such as involucrin and loricrin, but the role of filaggrin in this process is crucial (Montero-Vilchez et al., 2021). Keratinocytes are bound with protein fibers, corneodesmosomes that resemble steel rods within a brick structure. They are made of proteins of the cadherin family: mainly desmoglein, but also desmocollin and corneodesmosin. The whole forms a very durable, and at the same time flexible, protective structure characterized by dynamic processes related to the multiplication of cells, their death and exfoliation (Rajkumar and Chatterjee, 2023).

The production of a natural moisturizing factor in the skin, whose presence conditions the normal physiological processes that occur in the skin, is necessary to maintain the integrity of the epidermal barrier. Deamination of filaggrin occurs under the influence of peptide deiminase (Rajkumar and Chatterjee, 2023). Then, in subsequent processes it degrades to low-molecular-weight peptides and further to free amino acids.

In the right pH conditions (pH 5.5 for adult skin) they are catabolized to basic ingredients such as lactic acid, urocanic acid, sodium salt of pyrrolidine carboxylic acid and urea (Pretel-Lara et al., 2024). NMF hygroscopicity results in it being indispensable for water retention (its components absorb water and dissolve in it) inside the corneocytes, maintaining the correct volume. This mechanism prevents gaps between corneocytes. The epidermal barrier efficiency is determined by the value of the transepidermal water loss (TEWL) factor (Kundu et al., 2025).

### Skin reaction, skin pH

A very important factor affecting enzymatic processes participating in cell proliferation is the pH value. In healthy children after birth, its value is close to neutral, i.e., circa pH 6.5. After a few weeks it changes and reaches the level typical for adults, namely, pH 5.4–5.9. The pH value of the epidermis is influenced by endo- and exogenous factors, such as too high and low temperature, phospholipase A2, NMF components, sweat components, sebum, microbiota metabolites, or organic and inorganic chemical compounds applied to the skin (John et al., 2023). The efficiency of the epidermal barrier is also determined by the presence of serine and cysteine protease in the stratum corneum. Correct exfoliation of the epidermis is conditioned by the distribution of proteins within the corneodesmosomes, where serine proteases also regulate stratum corneum lipid synthesis. They degrade extracellular lipid-processing enzymes leading to a decrease in the secretion of stratum corneum lipids by lamellar bodies. The degradation of connections between corneocytes is also caused by the activity of cysteine proteases which in turn is regulated by the activity of the appropriate inhibitor - cystatin. In healthy epidermis, cystatin is also secreted on its surface together with sweat providing protection against exogenous proteases produced e.g., by demodex mites, house dust mites or golden staphylococci (Aiello et al., 2025). The functional balance of proteases and their stratum corneum inhibitors depends on the pH value on the surface of the skin. Acidification, by reducing the activity of LEKTI or serine protease inhibitor and intensifying the activity of serine protease itself, enhances surface peeling processes (Duffy et al., 2017). The natural process of proliferation and peeling of the skin, “the turnover” In the natural process of exfoliation of the epidermal cells, the key role is played by proteolytic degradation of desmosomes due to the activity of proteases and the deactivation of inhibitors. This process is also to a large extent dependent on the lipids of the intercellular matrix. The “turnover” exfoliation affects the maintenance of proper humidity with proper NMF synthesis and thus the pH value of the lipid coat. Epidermal replacement is a natural process that cleanses the skin of toxins, bacteria, and mite droppings; the rate of renewal decreases with age. In young people up to the age of 30, this process takes about 14 days, while in 50-year-olds it requires up to 37 days (Yan et al., 2023). As a result of prolonged exposure to extreme conditions in human skin, demodicosis can occur. It is a group of chronic inflammatory dermatoses of the skin, leading to weakening of the skin-epidermal barrier. It results from disturbances in the natural process of exfoliation and renewal of the epidermis, leading to a slower self-purification of sebaceous glands from demodex faeces (guanine and protease). The inflammatory reaction initially manifested by skin sensitivity to cosmetics and external factors is probably associated with high levels of guanine and proteases (Hong et al., 2025). Guanine, present in mite droppings, is a toxic compound and a strong allergen which is probably responsible for immunological reactions. Cysteine and serine stratum corneum proteases have also been observed in mite droppings, such as scabies, demodicosis and house dust mites (Yue et al., 2024). Their increasing activity and concentration within the sebaceous glands may have a direct impact on the coherence of the epidermal-lipid barrier. The association with increased activity of stratum corneum proteases and decreased activity of inhibitors such as cystatin is the result of increased epidermal pH (towards alkalization) and TEWL index, decreased ceramide concentration and increased concentrations of free fatty acids and sterols in lipids (Criado et al., 2024).

## Materials and methods

### Ethics

Written informed consent was obtained from the individual(s) for the publication of any potentially identifiable images or data included in this article. The study was conducted on 90 healthy volunteers exposed to different types of extreme environmental conditions. Participants were divided into three experimental groups (n = 30 per group) depending on the environmental conditions to which they were exposed. The exposure period lasted 4 weeks, and participants were exposed to the tested conditions for 8 h per day. The following environmental exposure groups were included:

1. Extremely dry air conditions simulating aircraft cabin environments.
2. Alternating high and very low temperatures (dry sauna followed by ice chamber exposure).
3. High humidity conditions.

All participants were adults with comparable age distribution and without diagnosed dermatological diseases. The study was conducted under dermatological supervision. The research protocol was approved by the Bioethical Committee, and all participants provided written informed consent before participation. Environmental parameters were precisely controlled for each group.

#### Extremely dry air

Participants were exposed to air with 10% relative humidity and temperature maintained at 22.5 °C, simulating conditions commonly encountered during long-haul flights in aircraft cabins.

### Alternating high and low temperatures

Participants were exposed to cyclic thermal stress consisting of alternating exposure to: dry sauna at 80 °C for 15 min and ice chamber at −10 °C for 15 min. These cycles were repeated several times during the daily 8-h exposure period.

### High humidity

Participants in the third group were exposed to steam sauna conditions with relative humidity between 65% and 80% and temperature of approximately 50 °C. The high humidity exposure was conducted in a steam sauna facility (Malta Thermal Baths, Poznań, Poland), where relative humidity ranged between 65% and 80% and temperature was maintained at approximately 50 °C.

### Participant characteristics

The study included 90 healthy volunteers (n = 90) divided into three groups of 30 participants each. The age range was 25–45 years (mean: 34.2 ± 5.8 years).

Inclusion criteria: absence of active dermatological diseases, no diagnosis of atopic dermatitis, psoriasis, or active acne, no systemic retinoid therapy within the last 6 months, no immunomodulatory treatment and no aesthetic dermatology procedures within the last 3 months.

Exclusion criteria: pregnancy or lactation, autoimmune diseases, diabetes mellitus, chronic dermatological disorders and smoking more than 10 cigarettes per day. All participants provided written informed consent prior to inclusion in the study.

### Standardization of measurement conditions

All measurements were performed under controlled environmental conditions: temperature: 22 °C ± 1 °C, relative humidity: 45% ± 5%, after a 20-min acclimatization period and between 8:00 a.m. and 11:00 a.m.

Participants were instructed: not to apply cosmetic products for at least 12 h prior to measurement and not to wash the face for at least 4 h before examination. Each parameter was measured three times, and the mean value of the three readings was used for statistical analysis.

### Instrumental measurements

Skin biophysical parameters were measured using non-invasive bioengineering methods with devices manufactured by Courage + Khazaka Electronic GmbH (Cologne, Germany).

The following parameters were evaluated:

Skin hydration by device: Corneometer CM 825. Measurement of the electrical capacitance of the stratum corneum, reflecting its water content. Units: arbitrary units.

Transepidermal water loss (TEWL) by device: Tewameter TM 300. Principle: open-chamber diffusion method measuring the rate of water evaporation from the skin surface. Units: g/m^2^/h.

Skin pH by device: Skin-pH-Meter PH 905. Measurement based on a flat glass electrode. Accuracy: ±0.1 pH units.

Sebum level by device: Sebumeter SM 815. Principle: photometric method measuring lipid absorption on a special plastic tape. Units: (a.u.).

Skin elasticity by device: Cutometer MPA 580. The device measures mechanical deformation of the skin under controlled negative pressure. Parameter analyzed: R2 (gross elasticity). Values range from 0 to 1, representing the elasticity coefficient.

### Sampling procedure

Two types of measurements were performed: blank sampling performed before the beginning of daily exposure to extreme environmental conditions and control sampling performed immediately after the 8-h exposure period. Measurements were taken from standardized facial areas, including: cheeks and T-zone to ensure comparability between participants. Newly developed diagnostic and care-oriented method. The methodological approach proposed in this study consists of a four-step diagnostic model enabling identification of skin barrier disturbances and selection of appropriate skincare strategies.

- Step 1 – Identification of environmental stressor
- Very dry air (<15% relative humidity)
- Cyclic temperature fluctuations (80 °C sauna/−10 °C ice chamber)
- High humidity exposure (65%–80%)
- Step 2 – Instrumental assessment of epidermal barrier function

Evaluation of the following parameters:

- Transepidermal water loss (TEWL) primary indicator of barrier integrity
- Skin pH indicator of enzymatic balance
- skin hydration reflecting natural moisturizing factor (NMF) status
- Sebum level
- Skin elasticity
- Step 3 – Interpretation of observed changes

Typical diagnostic patterns include:

- HighTEWL, lipid barrier impairment
- pH > 6.0, disturbance of protease–inhibitor balance
- Hydration <45 a.u., possible NMF deficiency
- Sebum <6 a.u., impaired sebaceous gland function
- Step 4 – Selection of targeted skincare strategy

Depending on environmental conditions:

- Dry environments, emollients rich in ceramides and occlusive agents
- High humidity, sebum-regulating formulations and antioxidants
- Cyclic thermal stress, barrier-repair formulations and pH normalization

This structured approach represents the core methodological contribution of the study and provides a clinically applicable diagnostic-care algorithm.

### Statistical analysis

Quantitative data were expressed as mean ± standard deviation (mean ± SD). Normality of distribution was verified using the Shapiro–Wilk test. Differences between groups were evaluated using one-way analysis of variance (ANOVA) followed by Dunnett’s post-hoc test for multiple comparisons. Statistical significance was set at α = 0.05. All statistical analyses were performed using Statistica 13.3 (TIBCO Software Inc.).

## Results and discussion

### Extremely dry air conditions (imitating air on an airplane)

In charts below averaged results of measurements of: skin hydration, transepidermal water loss (TEWL), skin pH value, skin lubrication and elasticity have been presented.

### Skin hydration level

**FIGURE 1:**
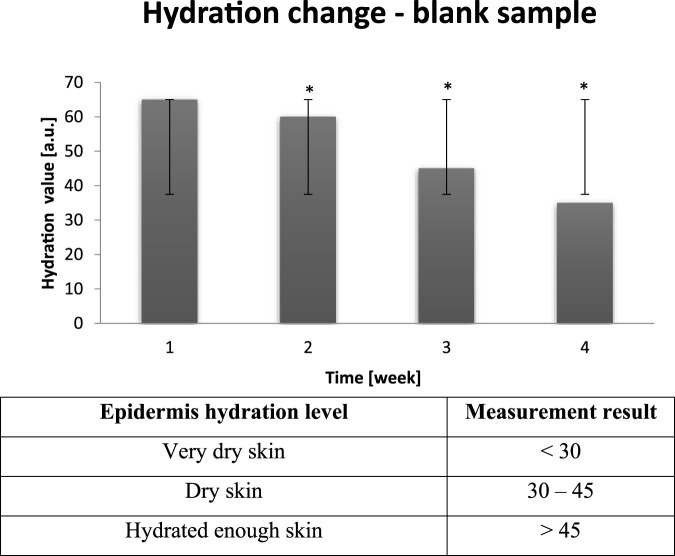
Averaged change of probants’ skin hydration calculated on the basis of the sampling after leaving a dry air area (n = 30). Error bars represent standard deviation (SD). *p < 0.05 vs. baseline (Week 1).

Average results of skin hydration level measurements conducted before the entry to a dry air area are presented in Figure 1. The results were averaged for 30 probants. All measurements were carried out on cheeks and in T-zones.

On the basis of Figure 1 it can be noticed that for all examined individuals hydration parameter was decreasing in time. Figure 1 shows that skin is a complex organ and, despite the negative external factors, spontaneously attempts to restore proper physico-chemical parameters. Changes of hydration parameter were in range 65–35 [a.u.].

**FIGURE 2:**
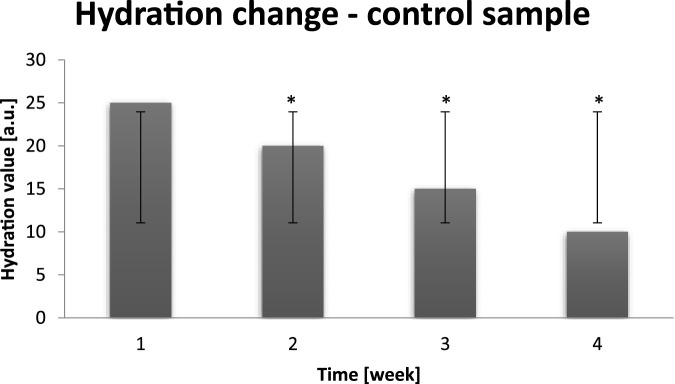
Averaged change of probants’ skin hydration calculated on the basis of the sampling after leaving a dry air area (n = 30). Error bars represent standard deviation (SD). *p < 0.05 vs. baseline (Week 1).

During control sampling all probants showed skin hydration level to be normal. It can be noticed that during the time between dry air exposure and normal conditions, hydrolipid barrier was able to rebuild. Nevertheless, the longer time of dry air exposure the protective barrier regenerates longer and in not able to fulfill its role properly. Mentioned negative trend can be observed in Figure 2. Next skin hydration measurement was performed after all day exposure to dry air.

After control sampling it was noticed that average value of hydration was equal to 25–10 [a.u.]. Dry air distort the composition of the protective coating and intercellular cement. On the basis of the conducted study it can be stated that hydrolipid coating, which constitutes main skin protection, after several hour exposure to dry air, undergoes attenuation, thus cannot fulfill its functions properly. It contributes to the hydration deterioration, and, in extreme cases, to dehyratation. The most efficient method of skin hydration is strengthening of the water loss inhibiting barrier. The reduction of water evaporation from epidermis is directly connected to the hydration increase. On the basis of conducted research, cyclic decrease in hydration parameter during the experiment can be confirmed (Kucharska et al., 2024).

### Transepidermal water loss level (TEWL)

**FIGURE 3:**
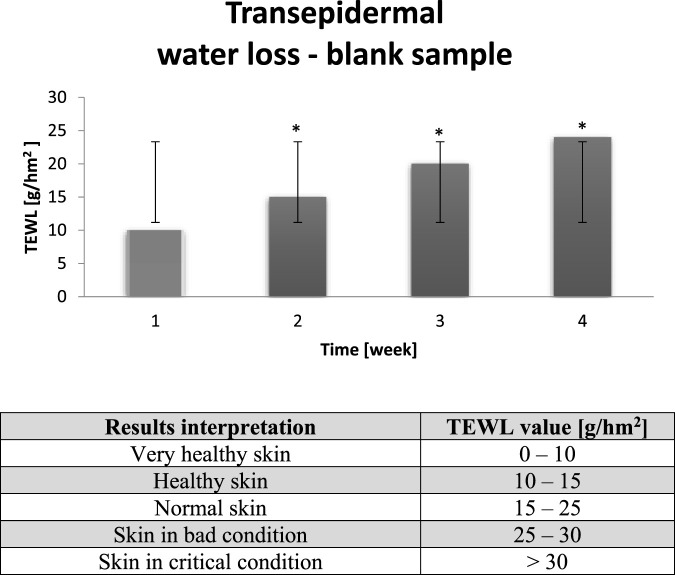
Averaged change of the TEWL value of probants based on sampling before entry to a dry air area (n = 30). Error bars represent standard deviation (SD). *p < 0.05 vs. baseline (Week 1).

The water retention ability of the stratum corneum is an important parameter regulating skin hydration. The dermis contains 80% of water, while the stratum corneum – only 13%. The skin appearance is conntected to a water retention by the epidermis. In chart below it can be observed that skin of probants tested towards the TEWL before several hour dry air exposure was characterized by proper transepidermal water loss level. All probants’ skin was healthy (Figure 3).

On the basis of Table 3 it can be noticed that skin is a complex organ and, despite the negative external factors, spontaneously attempts to restore proper physico-chemical parameters. On the basis of next figure it can be noticed that several hour dry air exposure was harmful for all probants (n = 30). Trensepidermal water loss value increased, which fact testifies to too fast water loss. The average TEWL value, measured before dry air exposure, depending on the experiment week, was equal to 10–25 [g/hm2]. It has to be underlined those good results of blank samples results from the lipophilic occlusive film formation, which is present until a detergent use. Due to systematic use of preparations (voluntarily chosen by probants for their home care), the occlusive action of lipid raw materials and physico-chemical effect on intercellular cement occurs (Khosrojerdi et al., 2024). Lipids builds in the cement structures, which allows to change the epidermal barrier properties (Liu et al., 2022). The tightness of occlusive film is dependent on the amount of lipid ingredients in cosmetic recipe and their chemical structure. When given lipid ingredient mixes with cement lipids, the direct influence on epidermal barrier is observed. The undirect influence occurs when the raw material changes the barrier properties only after its chemical transformation (Łoś-Rycharska et al., 2020). In the chart below results of control sampling after several hour dry air exposure is presented.

**FIGURE 4:**
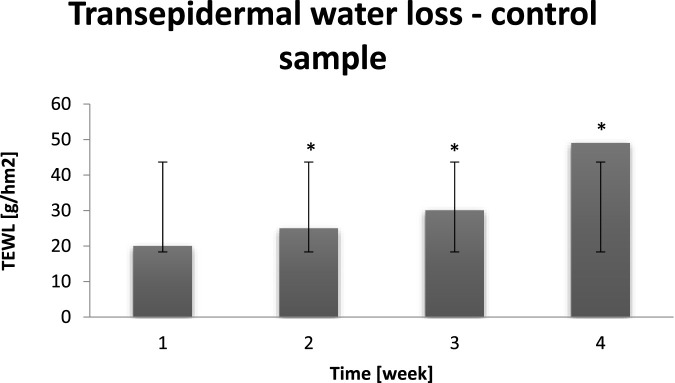
Averaged change of the TEWL value of probants based on sampling after leaving a dry air area (n = 30). Error bars represent standard deviation (SD). *p < 0.05 vs. baseline (Week 1).

The increase of TEWL on average by 20–40 g/hm^2^ during subsequent weeks of the experiment resulted in the excessive water evaporation, which led to decrease of the skin hydration and the negative internal factors resistance (Figure 4). The long term increase of this parameter results in the skin density deterioration and influences on the skin aging acceleration. It can be assumed that to enhance the skin condition and to inhibit the excessive water loss, preparations of occlusive action should be applied systematically (McAleer, 2023). Highly desirable components of these preparations are lipid raw materials, which provide probants’ skin protection against excessive transepidermal water loss. The TEWL value will decrease after such preparation’s application due to the phenomenon of merging/sticking of irregular cells, thus protective barrier increasing (Lim et al., 2024).

### Elasticity level

**FIGURE 5:**
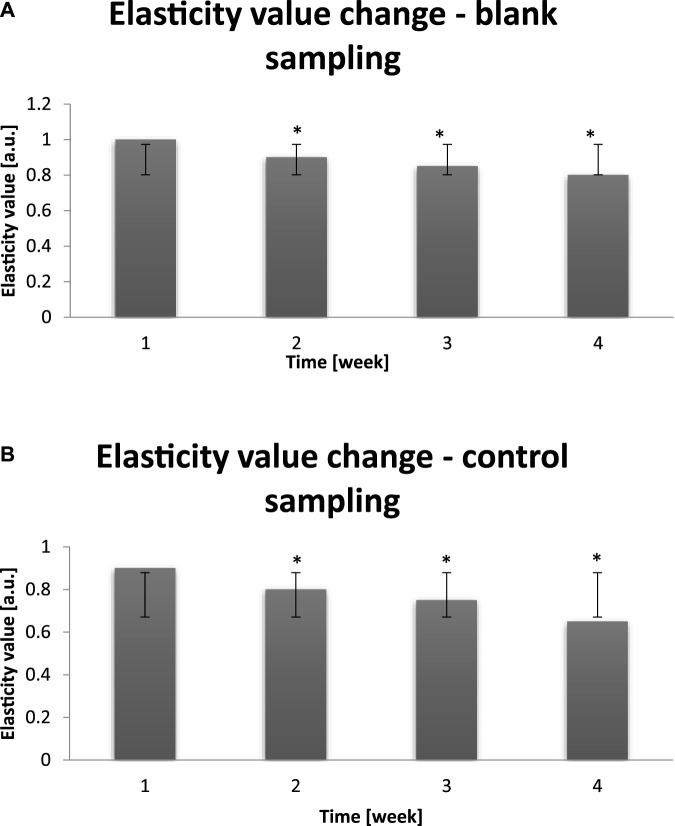
**(A)** Averaged change of the skin elasticity value of probants based on sampling before entry to a dry air area (n = 30). Error bars represent standard deviation (SD). *p < 0.05 vs. baseline (Week 1). **(B)** Averaged change of the skin elasticity value of probants based on sampling after leaving a dry air area (n = 30). Error bars represent standard deviation (SD). *p < 0.05 vs. baseline (Week 1).

In the chart below the next parameter – elasticity – is shown. Analogously, the parameter was examined before the entry to a dry air area on the group of 30 probants. Also analogously, measurments were performed on cheeks and in T-zones (Figure 5A).

The elasticity is a biomechanic feature of dermis, which is composed of connective compact tissue. The tissue includes collagen fibers and elastin fibers, which are responsible for the skin elasticity. During aging the natural proces of these fibers production undergoes deceleration. The synthesis and activity of fibroblasts is impared, which results in their functions distortion. The aftermath is the decrase in skin elasticity, which becomes flabby, dry, thinner and devoided of shine and vitality (Nicol et al., 2021). At this time, first wrinkles are formed, and the skin condition decreases significantly. External conditions and factors contribute to the deterioration of the examined parameter, which can be observed in Figures 5A,B. The average change of the elasticity, equal to 1–8 [a.u.], in the time of study was noticed.

The examination of probants’ skin elasticity confirmed that dry air exposure decreases the value of this parameter oscillating in between 0.85–0.65 [a.u.]. The interpretation of the epiderm elasticity measurement results is as follows: the value of the skin elasticity factor closer to 1 (100%) the more flexible skin is.

### Sebum level

**FIGURE 6:**
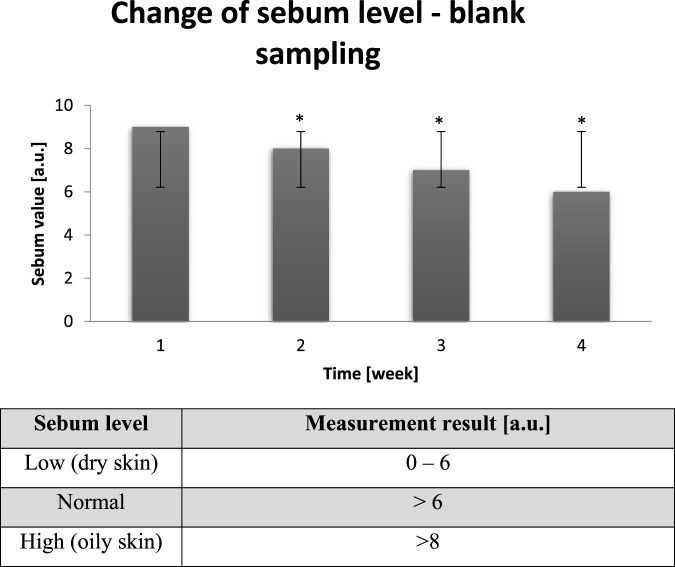
Averaged change of the sebum level of probants based on sampling before leaving a dry air area (n = 30). Error bars represent standard deviation (SD). *p < 0.05 vs. baseline (Week 1).

Next measured parameter clearly showed how significantly external conditions influence on probants’ skin and how particular parameters influence on each other. In the chart below results of probants’ (n = 30) sebum measurements after all day dry air exposure are presented (Figure 6).

**FIGURE 7:**
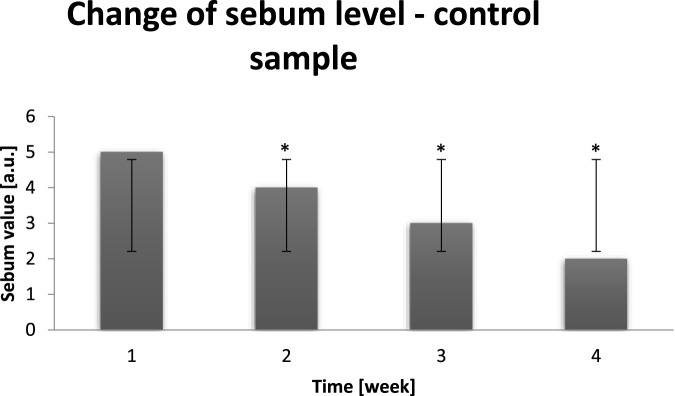
Averaged change of the sebum level of probants based on sampling after leaving a dry air area (n = 30). Error bars represent standard deviation (SD). *p < 0.05 vs. baseline (Week 1). The main reason of skin dryness is its barrier function disorder. Outer layer of skin is called stratum corneum. Cells of stratum corneum are connected to each other with intercellular cement. One of its components are lipids, which inhibits skin water loss. When the amount of lipids is too low, the skin looses water and becomes dry. The only way to improve this state is active and passive hydration with use of products, which strengthen demaged barrier function of the skin (Bradshaw et al., 2023).

The dry skin, such as this of probants having after staying in extremely dry conditions, is very sensitive to environmental factors. Due to the conditions sebaceous glands ceased the production of proper amount of sebum, necessary for adequate skin oiling. Indeed, lipids together with the properly secreted sebum palys role of skin protection against extrernal factors (Simpson et al., 2025). When the sebum level is too low, skin is more exposed to aggressive external conditions. Above mentioned assumptions found confirmation in Figure 7.

### pH value measurements

**FIGURE 8:**
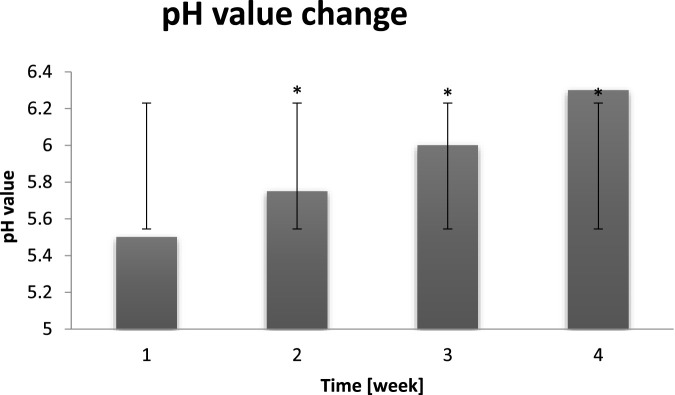
Averaged change of the skin pH value of probants based on sampling before entry to a dry air area (n = 30). Error bars represent standard deviation (SD). *p < 0.05 vs. baseline (Week 1).

Correct pH value of adult man should be in the range 5.4–5.9. This value is influenced by endo- and exogenous factors, such as too high or too low temperature, phospholipase A2, NMF components, sweat and sebum composition, microbiota metabolites, chemical organic and inorganic compounds applied to the skin (Hebert et al., 2020). The efficiency of epidermal barrier also depends on the presence of serine protease and cysteine protease of the stratum corneum. The proper exfoliation of the epidermis id dependend on protein distribution in corneodesmosomes, which is adjusted by serine protease regulated synthesis of the stratum corneum lipids. More information concerning the correct skin pH value maintenance importance can be found in literature description part (Vanessa et al., 2022). In Figure 8 below skin pH values of probants before dry air exposure is presented.

**FIGURE 9:**
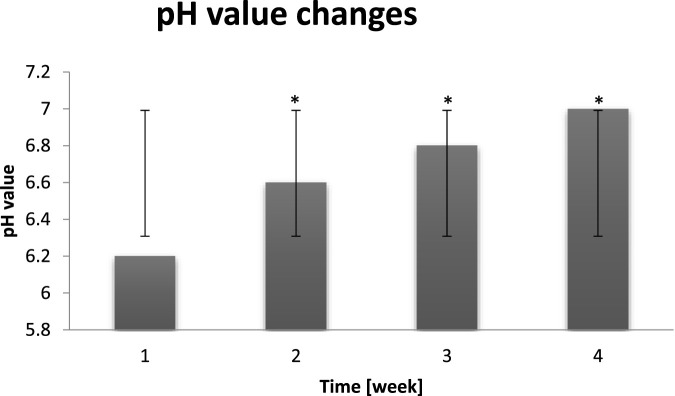
Averaged change of the skin pH value of probants based on sampling after leaving a dry air area (n = 30). Error bars represent standard deviation (SD). *p < 0.05 vs. baseline (Week 1).

The functional balance of proteases and their inhibitors of the stratum corneum is dependent on the skin pH value. The acidification as a result of LEKTI (serine protease inhibitor) activity reduction, causes surface exfoliation. Their increased activity and concentration in sebaceous glands can directly affect the cohesion of epidermis-lipid barrier. The increased activity of stratum corneum proteases together with the decreasing in activity of inhibitors, such as cystatin, is a result of the increase of pH value to alkaline range, TEWL level and free fatty acids and sterols concentration in lipids together with the decrease of ceramides concentration. As it can be seen in Figure 9, probants’ skin pH value oscillated in range of 5.5–6.3. The longer was the dry air exposure the higher was pH value, which is negative symptom indicating the hydrolipid barrier malfunction (Trzeciak et al., 2022).

**FIGURE 10:**
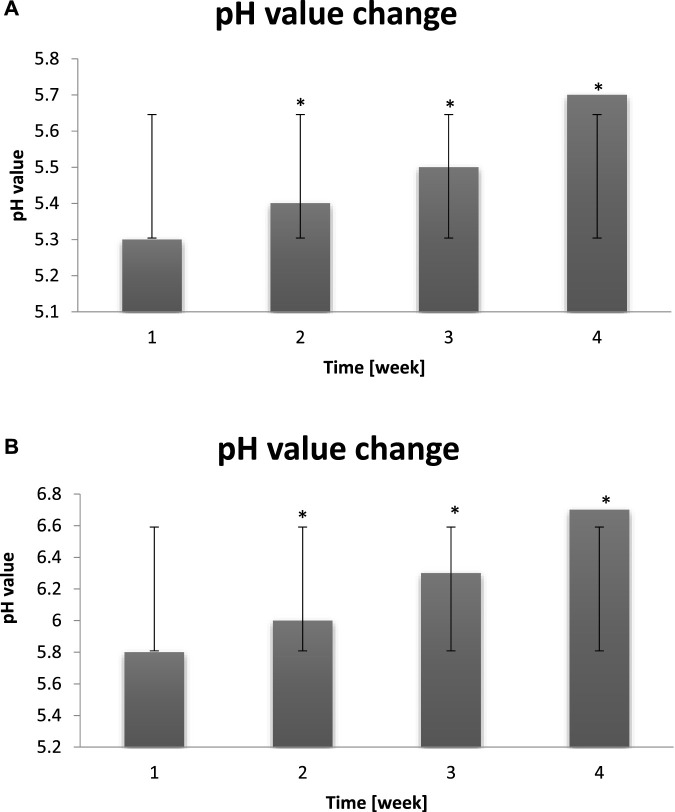
**(A)** Change of the probants’ skin pH value measured before exposure to “hot–cold air” conditions (n = 30). Error bars represent standard deviation (SD). *p < 0.05 vs. baseline (Week 1). **(B)** Change of the probants’ skin pH value measured after exposure to cyclic “hot–cold air” conditions (n = 30). Error bars represent standard deviation (SD). *p < 0.05 vs. baseline (Week 1).

The proper skin pH level is extremely important for its good condition. Both too acidic and too alkaline pH causes the dysregulation of intradermal processes and the barrier function impairment. If a face skin pH is too low, it produces more sebum, which results in the oily skin, blackheads and imperfections manifestation. On contrary, when pH is alkaline, skin becomes overdry and prone to irritation (Uchida and Park, 2021). On the basis of the chart it can be noticed that long term exposure to dry air conditions resulted in the pH value decrease to alkaline. As it can be seen in Figure 10 probants’ pH value oscillated in range of 6.2–7.0. The longer dry air exposure the higher pH value (pH may be alkaline) which is negative symptom, indicating hydrolipid barrier malfunction.

Hot and extremely cold air. (“Scandinavian”, “alpine” and “African” skin types)

Participants exposed to cyclic thermal stress (80 °C sauna/−10 °C ice chamber) were assessed using the same blank/control sampling scheme as described in Materials and Methods. Results are summarized in Tables 1–4.

**TABLE 1:** Change of probants’ skin hydration measured before and during the exposure to “hot–cold air” conditions.

Variable	Probants number (n)	Min	Max	Arithmetic average ( $\bar{x}$ )	Standard deviation (σ)	Relative error at 99% (δ)
Blank sample						
Initial hydration level	30	32,20	80,66	55,15	1,13	7,66
Hydration level after 1 week	30	29,50	65,98	47,00	0,90	7,12
Hydration level after 2 weeks	30	25,92	53,24	37,89	0,73	7,16
Hydration level after 3 weeks	30	19,78	49,10	35,20	0,83	8,78
Control sample						
Hydration level – control sample	30	32,12	71,54	51,05	1,11	8,09
Hydration level after 1 week	30	25,49	55,12	38,35	0,81	7,89
Hydration level after 2 weeks	30	24,58	54,29	37,53	0,81	8,06
Hydration level after 2 weeks	30	23,46	49,82	33,94	0,58	6,38

**TABLE 2:** Change of probants’ TEWL measured before and during the exposure to “hot–cold air” conditions.

Variable	Probants number (n)	Min	Max	Arithmetic average ( $\bar{x}$ )	Standard deviation (σ)	Relative error at 99% (δ)
Blank sample						
Initial TEWL level	30	6,20	13,50	9,58	0,23	8,99
TEWL level after 1 week	30	7,20	15,60	10,72	0,20	7,14
TEWL level after 2 weeks	30	7,80	17,70	11,80	0,22	7,14
TEWL level after 3 weeks	30	9,00	21,50	13,34	0,25	7,15
Control sample						
Initial TEWL level	30	7,80	17,70	11,80	0,22	7,14
TEWL level after 1 week	30	10,00	21,90	14,42	0,21	5,58
TEWL level after 2 weeks	30	10,90	33,20	19,49	0,50	9,59
TEWL level after 3 weeks	30	11,80	34,60	20,49	0,47	8,57

**TABLE 3:** Change of probants’ skin elasticity measured before and during the exposure to “hot–cold air” conditions.

Variable	Probants number (n)	Min	Max	Arithmetic average ( $\bar{x}$ )	Standard deviation (σ)	Relative error at 99% (δ)
Control sample						
Initial elasticity level	30	0,4643	0,8661	0,7588	0,0114	5,59
Elasticity level after 1 week	30	0,5596	0,8749	0,7725	0,0087	4,22
Elasticity level after 2 weeks	30	0,5702	0,8699	0,7787	0,0088	4,22
Elasticity level after 3 weeks	30	0,6312	0,8736	0,7913	0,0074	3,50
Blank sample						
Initial elasticity level	30	0,8101	0,9312	0,8722	0,0033	1,41
Elasticity level after 1 week	30	0,8207	0,9677	0,8889	0,0032	1,34
Elasticity level after 2 weeks	30	0,8362	0,9804	0,9044	0,0030	1,25
Elasticity level after 3 weeks	30	0,8532	0,9903	0,9134	0,0031	1,26

**TABLE 4:** Change of probants’ sebum level measured before and during the exposure to “hot–cold air” conditions.

Variable	Probants number (n)	Min	Max	Arithmetic average ( $\bar{x}$ )	Standard deviation (σ)	Relative error at 99% (δ)
Blank sample						
Initial sebum level	30	5	11	7,46	0,14	7,15
Sebum level after 1 week	30	3	10	6,82	0,14	7,79
Sebum level after 2 weeks	30	4	10	6,54	0,14	7,89
Sebum level after 3 weeks	30	0	10	5,85	0,21	13,45
Control sample						
Initial sebum level	30	3	10	6,82	0,14	7,79
Sebum level after 1 week	30	0	10	5,85	0,21	13,45
Sebum level after 2 weeks	30	0	9	4,64	0,22	18,01
Sebum level after 3 weeks	30	0	7	2,10	0,17	30,64

### Skin hydration level

In the table below results of skin hydration level measurements conducted before the entry to a dry sauna and ice chamber are presented. In Table 1 probants' epidermis hydration measurement results as a function of time are collected, including: hydration level variation range (Min. – Max.), arithmetic average ( $\bar{x}$ ), standard deviation (σ) and relative error at confidence level of 99% (δ). The results were obtained from blank sampling (before the entry to Malta’s Thermal Baths) and after all day cyclic stay in dry sauna (80 °C) and ice chamber (−10 °C). The measurements were performed before the start of the study and after 1–4 weeks of staying in extreme conditions.

On the basis of these results it can be concluded that the cyclic probants exposure to temperature changes causes the deterioration of skin hydration level. It should be stressed that all probants had enough skin hydration, even after 4 weeks of the study. Only during last week it was noticed that probants’ skin started to become dry (Table 1).

Hot dry air causes faster skin water loss. Probants’ skin became tight immediately. The cold air exposure of the skin boost this process additionally. A skin does not like rapid temperature changes and reacts to them with intensified “gymnastics” of blood vessles, which in the case of couperose skin results in erythema and increased susceptibility of blood vessels to rupture. When rapidly warmed, a skin evaporates water outside, which leads to its overdrying, and when cooled – reacts with “unsealing” of the protective barrier, which increases the risk of irritation caused by environment factors. The same trend can be observed during warm and cold season change. Due to the fact it is very important to choose home and professional skin care with taking into account the season of a year and its characteristic temperatures (Kahraman et al., 2019; Olejnik et al., 2023; Emilia et al., 2024).

### Transepidermal water loss level (TEWL)

Tables 1–4 present Min–Max ranges and descriptive statistics (mean, SD) for blank and control samples over the 4-week exposure period.

The results were obtained from blank sampling (before the entry to Malta’s Thermal Baths) and after all day cyclic stay in dry sauna (80 °C) and ice chamber (−10 °C). The measurements were performed before the start of the study and after 1–4 weeks of staying in extreme conditions.

On the basis of Table 2 it can be noticed that probants’ TEWL value after the temperature changes exposure deteriorated with the duration of the research. Initially healthy skin became normal skin (Table 2). The negative effect on the probants’ skin condition had also the thermal shock, which the skin was exposed to during the change from cold to hot area and *vice versa*. Inadequate care leads these blood vessels to rupture and skin reddening. In order to prevent excessive water loss and problems with blood vessels, the skin should be regularly moisturized and strengthened with preparations based on sealing substances. These cosmetics help to shrink, seal and strengthen blood vessels, thus protect them against cracking (Gardner et al., 2020).

### Elasticity level

The results were obtained from blank sampling (before the entry to Malta’s Thermal Baths) and after all day cyclic stay in dry sauna (80 °C) and ice chamber (−10 °C). The measurements were performed before the start of the study and after 1–4 weeks of staying in extreme conditions.

Heat and cold receptors perceive an increase or decrease in temperature only when the ambient temperature differs from the temperature of the skin’s surface. At the same temperature of the skin surface and the environment, the receptors are not stimulated. This state is called physiological zero (Mateu‐Arrom and Mora, 2025). The nerve pathway, which conducts impulses caused by the stimulation of heat and cold receptors, consists of four neurons. The I sensory neuron, which transmits excitation from the heat and cold receptors in the skin, is located in the spinal ganglia. Its protrusions enter the spinal cord through the dorsal roots and reach the sensory neuron II located in the posterior horns of the spinal cord. Axons go to the opposite side of the spinal cord and reach the thalamus in the brain, where the III sensory neuron is located in the posterolateral ventral nucleus. The IV sensory neuron is located in the medial gyrus of the cerebral cortex (sensory center). The conditions of changing temperature cause the loss of firmness, density and elasticity of the skin (Chazot et al., 2020). These changes result in the degradation of the skin’s supporting fibers, especially collagen, which contributes to the acceleration of the skin aging process. On the other hand, the supply of cooler air or water causes the barrier functions to be slower, the amount of intercellular lipids in the stratum corneum is reduced, and the symptoms of dryness increase. On the basis of the performed elasticity measurement, negative changes in the elasticity parameter were noticed. Initially, the skin elasticity of probants from 0.9 [a.u.]. With the duration of the study, a mean change of 0.8 [a.u.] was noticed. The skin’s elasticity coefficient is interpreted as follows - the closer this value is to 1 (100%), the more elastic the skin is.

### Sebum level

The results were obtained from blank sampling (before the entry to Malta’s Thermal Baths) and after all day cyclic stay in dry sauna (80 °C) and ice chamber (−10 °C). The measurements were performed before the start of the study and after 1–4 weeks of staying in extreme conditions.

As the temperature drops, we feel an intense cold on the skin. In one square centimeter of skin there are several times more cold receptors than heat receptors. Low temperature stimulates blood circulation and firms the facial muscles, but it should be remembered that the colder the slower the sebaceous glands work. The skin’s protective lipid coating becomes heavier and water is lost (Sukakul et al., 2024). The natural protective lipid layer of the skin becomes thinner than usual and does not sufficiently protect our skin against the influence of external factors. All this accelerates water loss, the skin becomes rough, dry and red, and in extreme cases it even starts to peel off. On contrary, high temperature stimulates the excessive work of the sebaceous glands. The temperature fluctuations that were induced in the probants caused the combination of both processes. There was a tendency to dry skin in the volunteers tested. Before the start of the study, all patients had normal skin (Botvid et al., 2023). The results fluctuated in the range of 2–6 [a.u.] after the control sample was performed. The longer the tests took, the drier the skin became and the sebaceous glands became unregulated. The results were interpreted on the basis of Table 4.

### Measurement of the pH value

The results were obtained from blank sampling (before the entry to Malta’s Thermal Baths) and after all day cyclic stay in dry sauna (80 °C) and ice chamber (−10 °C). The measurements were performed before the start of the study and after 1–4 weeks of staying in extreme conditions.

Measurements of the pH value of the blank sample oscillated between 5.2–5.7. The skin pH value is acidic. It varies slightly depending on the color of the skin (due to pigmentation), as people with dark skin tones have a slightly lower pH. The skin reaction also fluctuates depending on the time of day (Figure 10A). Changes are also noticeable seasonally - in summer it is slightly lower than in other seasons of the year. Each person can have individual values of this parameter (not everyone is exposed to the same weather conditions or detergents). The anatomical position of the skin also reflects the pH - where the skin is prone to perspiration or in contact with each other, the pH is more acidic (Lee et al., 2025). For example, on the forehead and cheeks it is more alkaline than in the armpits. This has been confirmed by the studies, the results of which are presented below. After the cycle of “warm-cold” conditions, the probants were characterized by the following pH values. The microbiome supports the skin protective functions. It protects the skin against allergens and pathogens, slows down aging and regulates the pH value. Beneficial microorganisms can be destroyed by not very gentle care, e.g., washing the face with aggressive detergents and irritating substances. As a result of non-delicate care, the skin balance may be disturbed, and the lack of a proper protective barrier makes the skin even more exposed to irritation from the cold causing dryness, redness on the face, tightness and discomfort. It is worth remembering that the main function of the skin is to protect the entire body against harmful external factors, and its negative condition in extreme cases may expose us to various types of serious diseases (US Food and Drug Administration, 2022). Alternating extreme heat and cold conditions increase the alkalinity of the pH. Too high pH (above 7) is associated with dryness, hypersensitivity and microbiota growth. The pH value of the skin decreases after washing the face with cold water, this aspect should be remembered when removing makeup from our skin every day (Figure 10B).

### High humidity (“Asian” skin type)

Mean values of hydration, TEWL, pH, sebum and elasticity are presented below.

### Skin hydration level

Based on the results, it can be seen that all the people who participated in the study had worse results of the moisturizing parameter as the experiment progressed. Changes in the moisturizing parameter of the control sample ranged on average in the range of 70–45 [a.u.]. On the other hand, after completing the sauna cycle, where the air humidity was 65%–80%, it can be noticed that the humidity deteriorated and amounted to an average of 30–45 [a.u.]. As can be seen, the function of the hydrolipid barrier is disturbed also in conditions of increased humidity.

### Transepidermal water loss level (TEWL)

Dry skin peels off, becomes rough and less elastic, which is not only a source of unpleasant sensations, but also higher sensitivity to cosmetics and various types of detergents. There is then burning, itching and redness. Due to its properties, the epidermis protects against excessive water loss and under certain environmental conditions the rate of water evaporation (TEWL) is established. This coefficient depends on the concentration of water in the environment, the concentration of water in the skin and the tightness of the epidermis barrier. During the tests performed as a control sample, this parameter fluctuated in the range The average TEWL value that was measured in the probants before skin exposure to humid air was 10–25 [g/hm^2^] depending on the study week. Exactly as in previous studies. An average increase in TEWL by 18–38 g/hm^2^ after consecutive weeks of measurements contributes to excessive water evaporation, which results in a decrease in skin hydration and a decrease in its resistance to negative internal factors. Dehydrated skin is not very flexible and resilient coating, which loses its firmness and tension. Due to the low moisture content of the skin, collagen and elastil structures are degraded, which leads to skin sagging and the formation of wrinkles (Darlenski et al., 2009; Darlenski and Fluhr, 2012; Klimas et al., 2023).

### Skin elasticity level

Another measured parameter is presented below - elasticity. Similarly, the parameter was tested on a group of 30 test persons before entering the room with humid air. Measurements were also made analogously on the cheeks and the T-zone. As already mentioned, the conditions and external factors contribute to the deterioration of the tested parameter, which can be seen on the basis of the results. Along with the duration of the study, there was a mean change equal to 1–0.8 [a.u.] (blank sample).The tests of measuring skin elasticity of the testees confirmed that humid air reduces the value of this parameter. It oscillated on average in the range of 0.75–0.55 [a.u.] - control sample. The interpretation of the epidermal elasticity level results is as follows: skin elasticity coefficient - the closer this value approaches 1 (100%), the more elastic the skin is. Too high a level of air humidity makes the skin dry, irritated, tense, red, and even less resistant to infections. Additionally, enlarged pores become visible, there may be a burning sensation and peeling of the skin. As a result, it begins to age faster and the expression lines appear more noticeable (Dweck, 2021).

### Sebum level

Another measured parameter showed to a great extent how the conditions in which we stay affect our skin. The values of the sebum measurement of the probants (n = 30) are presented below, similarly before and after spending the whole day in humid conditions. Every day our skin has important mechanisms that protect it from the external environment and allow it to stay healthy and in good condition. One of these processes is the production of sebum. Sebum is a natural secretion produced by the sebaceous glands in the dermis. It is composed of a mixture of lipid substances such as glycerides, waxes, squalene and cholesterol. The amount of sebum our skin produces depends, among other things, on the testosterone metabolite - DHT. Sebum is produced in the greatest amount in the case of: oily, mixed, problematic and excessively dry skin. The mixture of lipids is part of a specific protective barrier of the epidermis that protects it against external factors: pathogens, UV radiation, injuries, frequent changes in temperature, chemical hazards and loss of moisture from cells. It is thanks to sebum that the skin can maintain the correct pH value and does not lose too much water from its structure (Coderch et al., 2021). When there is too little of it, the skin becomes clearly: dull and devoid of healthy glow, rough, unpleasant to the touch, thin and slack. In the tested conditions of too high humidity, it was noticed that the subjects in the blank sampling had a sebum value in the range of 6–8 [a.u.], which is interpreted as correct. After subjecting the patients to high humidity, it was noticed that this parameter was increased, which indicates excessive sebum secretion under the influence of the tested condition (9–12 a.u.). The overproduction of sebum can lead to serious skin problems. As a result of the long-term excess of sebum, imperfections and inflammations may appear on the skin. It is an excellent breeding ground for pathogenic bacteria that live on our face. During the digestion of sebum, microorganisms break down triglycerides into fatty acids, which in turn are the cause of the increased work of the sebaceous glands (Zolotareva et al., 2023). An anaerobic environment is also created on the skin, especially in the pores. With excess sebum, they become strongly clogged, leading to the formation of blackheads and painful eruptions (especially in the so-called T-zone - forehead, nose and chin). As a result, the complexion not only glows unsightly, but also problems with imperfections and acne begin to appear. One of the causes of excessive activity of the sebaceous glands is the already mentioned vicious cycle - triglycerides broken down into fatty acids stimulate even greater production of sebum (Im et al., 2021). However, this is not the only factor that may affect the increased secretion of sebum. The hormone testosterone is also responsible for this. This is where, for example, men’s skin is more prone to oiliness. In turn, estrogen and progesterone inhibit the work of the glands, which is why, for example, pregnant women usually do not complain about the shine of the face. So it’s worth remembering that men’s and women’s skin may require slightly different care (Kammeyer et al., 2022). The other causes of excessive sebum production include: age - the biggest problem appears in adolescence, genetic conditions, excessive and prolonged stress, improper diet low in vitamins and full of processed and fast food, strong air pollution and weather conditions, too frequent touching the face with hands, some medications, especially those based on hormones and steroids, highly comedogenic preparations - clogging pores, using cosmetics that excessively dry the skin (Choi and Ahn, 2023). We should eliminate as many causes of the increased activity of sebaceous glands as possible - introduce a diet rich in vitamins and unsaturated fatty acids, avoid processed and unhealthy foods, try to live in harmony, without stress and remove cosmetics that dry the skin from care. This can be difficult to implement, especially in the early stages. However, systematic implementation of changes in life will result in less glare on the forehead, nose and chin. Unfortunately, with some causes we can’t handle that easily. These are primarily genetic determinants and the environmental conditions in which we live. Then it is also worth reaching for proven care methods that combat excessive sebum production and those that do not remove the cause, but the effect. Let us not dry the skin, especially in summer and winter seasons). Wash your face with lukewarm or warm water. We should also try to cleanse the skin up to two times a day so as not to expose the skin to friction. This will reduce the occurrence of irritation. We should also remember that due to excessive care (e.g., repeated washing or rubbing with a tonic during the day), the skin may start to defend itself and produce more sebum. We should also remember to hydrate the body from the inside - drink 1.5–2 L of water regularly every day. However, we should avoid sweetened drinks full of artificial colors. Let’s try to provide ourselves with the right amounts of vitamins. Cigarette smoke can also contribute to the excessive work of the sebaceous glands (Rousseau et al., 2021).

### pH value measurement

The pH value is a very important parameter measured before and after the exposure.

**FIGURE 11:**
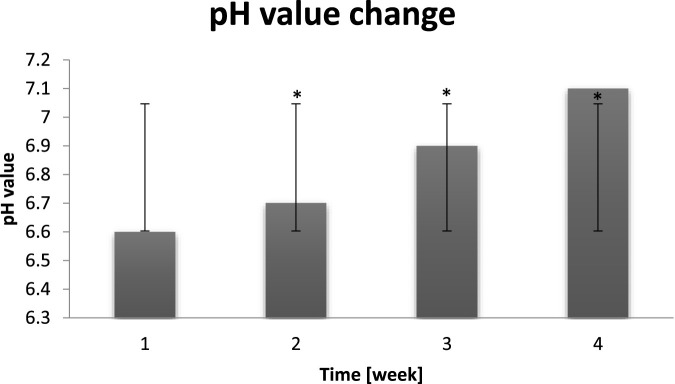
Change of the pH value of the probants measured before and during exposure to increased humidity (n = 30). Error bars represent standard deviation (SD). *p < 0.05 vs. baseline (Week 1).

The correct pH of the skin is extremely important for keeping it in good condition. Both too acidic and alkaline pH affects the dysregulation of the processes taking place in it and disrupts the barrier functions. If the pH of the facial skin is too low, it begins to produce more sebum, which can lead to oily skin, blackheads and imperfections. Conversely, if the pH is alkaline, the skin becomes dry and prone to irritation. The acidic pH of the skin is the result of the physiology of the human body, which in turn regulates the growth of the natural and desired microbiota. Human epidermis shows a slightly lower body temperature, slightly acidic pH and is quite dry, therefore it is not a friendly environment for most bacteria, which prefer a temperature of 37 °C, high humidity and neutral pH (within 7.0) (O’Regan and Irvine, 2020). Some skin microorganisms play an active role in maintaining an acidic pH and control the growth of other strains by secreting so-called bacteriocins (antibiotics that destroy unwanted bacteria). This prevents colonization by other pathogenic bacteria. On the basis of the conducted research, it was noticed that the probants, who in the control sample had the correct pH values oscillating in the range of 5.3–5.5, experienced its significant increase after being in high humidity conditions, as shown in the diagram below (Figure 11).

According to research, altered skin pH is associated with extreme humidity and temperature conditions, dermatological problems, such as contact dermatitis, atopic dermatitis, ichthyosis, *Candida* albicans infections and acne. It is also known that external factors such as soaps, skin care cosmetics, protective cosmetics, antibiotics or antiseptics applied to the skin significantly change the pH of the skin. The alkaline pH facilitates the spreading of bacteria over the entire surface of the epidermis, promotes the development of pathogenic strains, and also promotes the growth of bacteria that contribute to the unpleasant odor of sweat. The surfactants in hand soaps, shower gels and shampoos most often contribute to increasing the pH. Labels begin to show information about the pH of the cosmetic, so we can choose the right product for us. Remember that cleansing products should always be washed with water and then toned with cosmetics to restore the proper pH of the skin. The composition of the sweat, i.e., the secretions of the sweat glands, is also important (Visscher et al., 2022). It consists mainly of water (about 98%), salt (NaCl - 0.6%–0.8%), urea, uric acid, ammonia (an increase in this compound increases the pH value of the skin), lactic acid, carbohydrates and minerals (containing potassium, calcium, magnesium and iron). Its composition depends primarily on whether it is sweat secreted “interrupting” (i.e., physiologically without increased body temperature regulation; periodic secretion - some glands secrete while others remain inactive) or secreted “constantly” (i.e., during a period of increased physical activity, extreme conditions or increased stress). The concentration of compounds in sweat increases with periodic release due to reabsorption of water in the discharge tubules. It also depends on the food consumed, climatic conditions, hormonal factors and coexisting diseases. Sweating is a constant process - under conditions of rest and thermal comfort, we release a small amount of sweat (the so-called “invisible” sweating). About 5% of the eccrine glands work then and a trace amount of the apocrine glands. Sweating intensifies after the action of a thermal stimulus (the so-called “thermal” sweating) or an emotional stimulus (the so-called “emotional” sweating). Sweat is secreted by the sweat glands with the help of sweat pores, which are classified as skin appendages. They are located throughout the entire skin (with the exception of the glans penis, the inner part of the foreskin, the labia minora, the lower part of the labia majora and the nail matrix), but most of them are located in the skin of the hands and feet (Visscher et al., 2022).

## Conclusion

The present study demonstrated that prolonged exposure to extreme environmental conditions significantly impairs epidermal barrier function. Regardless of the type of environmental stressor, consistent changes in key skin parameters were observed. A decrease in skin hydration and elasticity accompanied by a significant increase in transepidermal water loss (TEWL) indicated progressive disruption of the hydrolipid barier (Vanessa et al., 2022; Trzeciak et al., 2022; Uchida and Park, 2021; Kahraman et al., 2019; Olejnik et al., 2023; Emilia et al., 2024; Gardner et al., 2020; Mateu-Arrom et al., 2025; Chazot et al., 2020; Schild et al., 2024). Exposure to very dry air and cyclic thermal stress resulted in reduced sebum secretion and a shift of skin pH toward alkaline values. In contrast, high humidity conditions increased sebum production but were also associated with elevated TEWL and decreased skin elasticity. These results indicate that both low and high humidity environments may destabilize epidermal barrier homeostasis (Duffy et al., 2017; Yan et al., 2023; Hong et al., 2025; Yue et al., 2024; Criado et al., 2024; Kucharska et al., 2024; Hansen-Sackey and Hartono, 2025; Abdel-Mageed, 2025; Nomura and et a l., 2021; Chu et al., 2024; Davis et al., 2023; Europ ean Task Force on Atopic Eczema, 2025). The proposed diagnostic approach based on instrumental assessment of hydration, TEWL, pH, sebum level, and elasticity enables early detection of barrier dysfunction and supports the selection of targeted skincare strategies. Proper skincare focused on lipid barrier restoration, pH normalization, and reduction of transepidermal water loss appears essential for maintaining skin homeostasis under extreme environmental conditions (Sukakul et al., 2024; Botvid et al., 2023; Lee et al., 2025; US Food and Drug Administration, 2022; Darlenski et al., 2009; Darlenski and Fluhr, 2012; Klimas et al., 2023; Dweck, 2021; Coderch et al., 2021; Zolotareva et al., 2023; Im et al., 2021; Kammeyer et al., 2022; Choi and Ahn, 2023; Rousseau et al., 2021; O’Regan and Irvine, 2020; Visscher et al., 2022; Rigano et al., 2021).

### Practical implications

Based on the obtained instrumental measurement results and the analysis of the mechanisms underlying epidermal barrier function, the following practical conclusions have been formulated regarding skincare for individuals exposed to extreme environmental conditions:

Rebuilding the hydrolipid barrier. Extreme temperatures and low humidity cause a significant increase in TEWL and damage to the intercellular lipid matrix. Recommendation: the use of emollients rich in ceramides, cholesterol and fatty acids to reduce water loss and restore barrier integrity. Normalization of skin pH. The observed increase in pH to alkaline values requires the use of products with a physiological pH. Recommendation: choosing dermocosmetics with a pH of 5.0–5.5 and avoiding cleansing agents with strong degreasing and alkalizing effects. Introduction of antioxidants. Exposure to extreme temperatures promotes oxidative stress. Recommendation: the use of products containing vitamin C, vitamin E or polyphenols to reduce oxidative damage. Regulation of the desquamation process. Extreme conditions disrupt natural exfoliation. Recommendation: gentle exfoliation (PHA, mild AHA) instead of aggressive peels, especially during exposure to extreme temperatures and humidity. Individualization of skincare depending on environmental conditions. The study demonstrated different patterns of change depending on the climate.

Recommendation:

- in dry air – intensive occlusion and lipid replenishment,
- in high humidity – sebum regulation and soothing of irritation,
- during temperature fluctuations – barrier protection and regeneration after exposure to so-called thermal shock.

Replenishing the NMF (Natural Moisturizing Factor). A decrease in hydration and indirect signs of filaggrin degradation justify NMF supplementation. Recommendation: the use of humectants (glycerin, PCA, amino acids, urea 3%–5%). Modification of daily skincare routines. Recommendation: avoiding alcohol-based cosmetics, strong detergents and intensive exfoliating treatments; using barrier creams, lipid-rich balms and TEWL-reducing products. Importance of instrumental assessments. Recommendation: regular evaluation of TEWL, hydration and pH parameters in individuals working or living in extreme environmental conditions to detect early signs of barrier impairment and select effective skincare strategies.

### Strengths

The present study has several important strengths. First, it provides a comprehensive and multidimensional assessment of skin response to extreme environmental conditions. Multiple objective, instrumental measurements were performed, including skin hydration (corneometer), transepidermal water loss (TEWL; tewameter), pH measurement, sebum level (sebumeter), and elasticity assessment. The simultaneous evaluation of these parameters allowed for an integrated analysis of epidermal barrier function. Second, the study design included exposure to three different types of extreme conditions: very dry air, cyclic high–low temperature changes, and high humidity. This approach enabled comparison of distinct environmental stressors within a uniform methodological framework. Third, the 4-week observation period allowed for monitoring dynamic changes over time rather than relying solely on short-term responses. Statistical analysis (ANOVA with Dunnett’s test) further strengthened the reliability of the results. Finally, the study was conducted in accordance with ethical standards and approved by the appropriate Bioethical Committee, ensuring methodological transparency and compliance with research regulations (Tang et al., 2025).

## Data Availability

The original contributions presented in the study are included in the article/supplementary material, further inquiries can be directed to the corresponding author.
